# Complete chloroplast genome sequences of two species used for Tibetan medicines, *Meconopsis punicea* vig. and *M. henrici* vig. (Papaveraceae)

**DOI:** 10.1080/23802359.2019.1693918

**Published:** 2019-12-11

**Authors:** Yixuan Zhu, Dequan Zhang

**Affiliations:** a College of Pharmacy and Chemistry, Dali University, Dali, China;; b Institute of Materia Medica, Dali University, Dali, China;; c Key Laboratory of Yunnan Provincial Higher Education Institutions for Development of Yunnan Daodi Medicinal Materials Resources, Kunming, China

**Keywords:** *Meconopsis*, complete chloroplast genome, Papaveraceae, phylogeny, Tibetan medicines

## Abstract

*Meconopsis* Vig. is a genus possessing important medicinal and ornamental values in the Papaveraceae. Many species in this genus are commonly used in traditional Tibetan medicines over thousands of years. In this study, we sequenced complete chloroplast (cp) genome sequences of two species, namely *Meconopsis punicea* and *M. henrici* to investigate their phylogenetic relationships in Papaveraceae. Total lengths of the chloroplast genomes were 153,281 bp and 153,388 bp, respectively. Both of the two genomes had typical quadripartite structure, LSC region (83,999 bp and 83,698 bp) and SSC region (17,730 bp and 17,822 bp) were separated by a pair of IRs (25,776 bp and 26,107 bp), respectively. Moreover, they were composed of 112 genes, including 78 protein coding genes, 30 tRNA genes, three rRNA genes and one pseudogene. Phylogenetic analysis based on complete chloroplast genomes showed that *M. henrici* had closer relationship with *M. racenosa* than *M. punicea*; meanwhile, *Meconopsis* was closely related to *Papaver* in Papaveraceae.

The genus *Meconopsis* Vig. belongs to the family Papaveraceae, comprises about 49 species in the world (Zhang and Grey-Wilson 2008). Most of the species in this genus are distributed in eastern Asia and Western Europe (Wu et al. 2011). Many species are well-popular by local people due to their economic and ornamental values with blue flowers, such as *M. grandis*, *M. racemosa*, and *M. horridula*; thus, they are figuratively called “Himalayan blue poppies”. However, some others, such as *M. lancifolia*, *M. quintuplinervia*, *M. punicea*, and *M. integrifolia*, have purple, red, or yellow flowers (Figure 1) (Guo et al. 2016). Some species of them have been widely used in traditional Tibetan medicine to treat inflammation, pain etc. (Wu et al. 2011). However, for such an important genus, most of the studies focused on their chemical compositions (Yang et al. 2010; Gao et al. 2013); moreover, although there were some studies on molecular biology (Ying et al. 2006; Qu et al. 2018), there was nearly no report of complete chloroplast genome of *Meconopsis* species, except *M. racemose* (Zeng et al. 2018). Here, we reported the complete chloroplast genome sequences of two important species, namely *M. punicea* and *M. henrici*, so as to reveal phylogenetic relationships between these species and related group in Papaveraceae.

**Figure 1.**
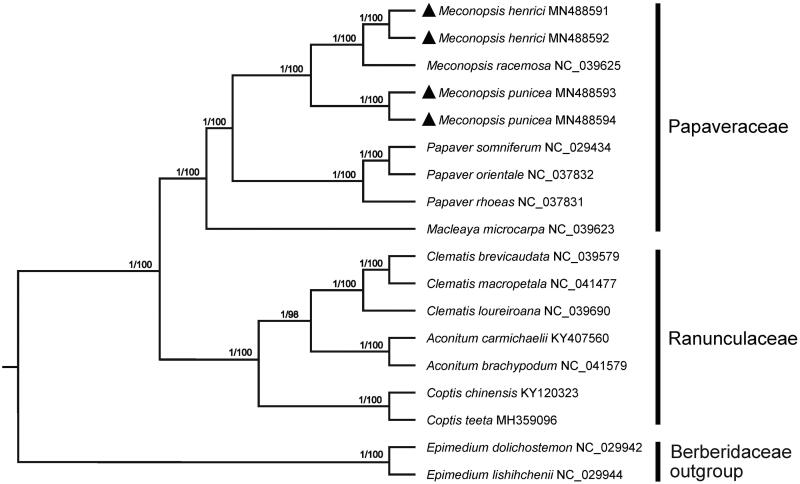
Phylogenetic tree of 14 species in Papaveraceae and Ranunculaceae constructed by complete chloroplast genomes with two Berberidaceae species as outgroups. Numbers above each branch represent the posterior probabilities obtained from the Bayesian Inference (before the slash) and the bootstrap values obtained from the Neighbor-Joining analysis (after the slash).

Fresh and clean leave materials of *M. punicea* and *M. henrici* were collected separately from Hongyuan county (N32°43′05.99″, E102°34′03.57″) and Litang county (N29°29′04.73″, E100°13′40.35″) in Sichuan province, China. Meanwhile, two voucher specimens with flowers (ZDQ17155 and ZDQ17123) were collected and deposited at the Herbarium of Medicinal Plants and Crude Drugs of the College of Pharmacy and Chemistry, Dali University. Total genomic DNA were extracted from fresh leaves by using the improved CTAB method (Doyle 1987; Yang et al. 2014) and genome sequencing were performed using HiSeq2000 (Novogene, Tianjin, China) platform with pair-end (2 × 300 bp) library. The raw data was filtered using Trimmomatic v.0.32 with default settings (Bolger et al. 2014). Then paired-end reads of clean data were assembled into circular contigs using GetOrganelle.py (Jin et al. 2018) with the cp genome of closely related species *Meconopsis racemosa* (NC_039625) as the reference. Finally, the assembled cp genomes were annotated and adjusted manually using Geneious 8.0.2. (Matthew et al. 2012). The annotated chloroplast genomes of *M. punicea* and *M. henrici* were submitted to the GenBank under the accession number of MN488593, MN488594 and MN488591, MN488592.

Total lengths of the chloroplast genome sequence of *M. punicea* and *M. henrici* were 153,281 bp and 153,388 bp, respectively. Among them, for *M. punicea*, a total of 36 SSRs were identified, a pair of inverted repeats (IRs) of 25,776 bp was separated by a small single copy (SSC) region of 17,730 bp and a large single-copy (LSC) region of 83999, for *M. henrici*, a total of 23 SSRs were identified, consisting of LSC region of 83,698 bp, SSC region of 17,822 bp, and IRs of 26,107 bp. They have a typical quadripartite structure, with the same 38.5% overall GC content. Both *M. punicea* and *M. henrici* cp genome contained 112 genes, including 78 protein coding genes, 30 tRNA genes, three rRNA genes and a pseudogene.

To investigate phylogeny of *M. punicea* and *M. henrici*, 16 plastomes belonging to Papaveraceae and related family were downloaded from GenBank and aligned using MAFFT v7.149 (Kazutaka and Standley 2013), the jModelTest v.2.1.7 (Darriba et al. 2012) was used to determine the best-fitting model (G + I+R) for the dataset. Then Bayesian inference (BI) was performed by MrBayes v.3.2.6 (Ronquist et al. 2012) and Neighbor-joining (NJ) tree was constructed using MEGA 7.0 (Kumar et al. 2016) with 1000 bootstrap replicates. Phylogenetic analysis based on complete chloroplast genomes showed that *M. henrici* had closer relationship with *M. racenosa* than *M. punicea*; meanwhile, *Meconopsis* was closely related to *Papaver* in Papaveraceae (Figure 1). We believe that the chloroplast genomes of *M. punicea* and *M. henrici* would provide valuable data for studies on species identification, molecular phylogenetics, and conservation genetics of *Meconopsis* and related genus in Papaveraceae.
